# Clinical efficacy of probiotics in the treatment of alcoholic liver disease: a systematic review and meta-analysis

**DOI:** 10.3389/fcimb.2024.1358063

**Published:** 2024-03-12

**Authors:** Shi-Ying Xiong, Gui-Sheng Wu, Chun Li, Wenzhe Ma, Huai-Rong Luo

**Affiliations:** ^1^ State Key Laboratory of Quality Research in Chinese Medicine, Macau University of Science and Technology, Macao, Macao SAR, China; ^2^ Integrated Traditional Chinese and Western Medicine Department, Yibin Sixth People’s Hospital, Yibin, Sichuan, China; ^3^ Key Laboratory for Aging and Regenerative Medicine, Department of Pharmacology, School of Pharmacy, Southwest Medical University, Luzhou, Sichuan, China; ^4^ Central Nervous System Drug Key Laboratory of Sichuan Province, Luzhou, Sichuan, China

**Keywords:** alcoholic liver disease, probiotics, intestinal flora, inflammation, meta-analysis

## Abstract

**Objective:**

Alcoholic liver disease (ALD) is a liver damage disease caused by long-term heavy drinking. Currently, there is no targeted pharmaceutical intervention available for the treatment of this disease. To address this, this paper evaluates the efficacy and safety of probiotic preparation in treating ALD through conducting a meta-analysis, and provides a valuable insight for clinical decision-making.

**Methods:**

A systematic search was conducted across databases, including PubMed, Embase, Web of Science, Cochrane Library, CNKI, VIP, Wanfang, and CBM from the inception dates to October 15, 2023, to identify clinical randomized controlled trials on probiotic preparations in the treatment of ALD. After the literature underwent screening, data extraction, and quality assessment, RevMan 5.3 and Stata 14.2 were employed for data analysis and processing.

**Results:**

A total of 9 randomized controlled trials fulfilled the inclusion criteria. The results of the meta-analysis showed that probiotic preparation could significantly improve the liver function of patients with alcoholic liver disease compared with the control group. Probiotic intervention led to a significant reduction in the levels of alanine aminotransferase (MD=-13.36,95%CI:-15.80,-10.91;*P*<0.00001),aspartate aminotransferase (MD=-16.99,95%CI:-20.38,-13.59;*P*<0.00001),γ-glutamyl transpeptidase (MD=-18.79,95% CI:-28.23,-9.34; *P*<0.0001). Concurrently, the level of serum albumin (MD=0.19,95% CI:0.02,0.36;*P*=0.03) was increased. Furthermore, probiotic intervention could also modulate the composition of intestinal flora in patients with alcoholic liver disease, leading to an augmentation in *Bifidobacteria* and a reduction in *Escherichia coli.* However, in patients with alcoholic liver disease, probiotic intervention showed no significant effects on total bilirubin (MD=-0.01,95% CI:-0.17,0.15;*P*=0.91), tumor necrosis factor-α (MD=0.03,95% CI:-0.86,0.92;*P*=0.94) and interleukin-6 (MD=-5.3,95% CI:-16.04,5.45;*P*=0.33).

**Conclusion:**

The meta-analysis indicates that probiotics can improve liver function in alcoholic liver disease, reduce inflammatory responses, regulate intestinal flora, which have potential value in the treatment of alcoholic liver disease.

**Systematic review registration:**

https://www.crd.york.ac.uk/prospero/, identifier CRD42023472527.

## 1 Introduction

Alcoholic liver disease (ALD) is a chronic liver disease caused by prolonged and excessive alcohol consumption, leading to various manifestations such as alcoholic fatty liver, alcoholic hepatitis (AH), and alcoholic cirrhosis. In severe cases, it can progress to liver failure or even mortality (Singal et al., 2018). A study conducted in the United States revealed that the increase in cirrhosis mortality was primarily driven by alcoholic cirrhosis, especially among people aged 25 to 34 (Tapper and Parikh, 2018). ALD is considered the cause of nearly half of liver related deaths worldwide (Mellinger et al., 2018). According to the 2018 Global Alcohol Status Report released by the World Health Organization, the mortality rates of AH and cirrhosis are particularly high, with a mortality rate of up to 50% for severe acute AH (World Health Organization, 2018). Therefore, the issue of alcohol-related liver disease has garnered increasing global attention. Alcohol and its metabolites exert detrimental effects on the liver through mechanisms including direct injury and oxidative stress (Galicia-Moreno et al., 2016). Genetic, environmental and epigenetic factors influence the progression of ALD to more severe forms (Argemi et al., 2020). Current treatment strategies for ALD encompass active abstinence, nutritional support, vitamin supplementation, and other general measures, pharmacological interventions may involve the use of steroids, pentoxifylline, and antioxidants (Siddiqi et al., 2020). However, substantial clinical evidence remains insufficient (Singal et al., 2018).

Numerous studies have demonstrated a strong correlation between the pathogenesis of ALD and alterations in the intestinal microbiota (Llopis et al., 2016; Bajaj, 2019; Ranjbarian and Schnabl, 2023). Prolonged alcohol consumption disrupts the composition of the intestinal microbiota, leading to increased levels of intestinal endotoxin, compromised intestinal barrier function, and subsequent translocation of bacteria and endotoxins from the portal vein to the liver, ultimately resulting in hepatic inflammation (Bull-Otterson et al., 2013). As beneficial microorganisms for hosts, probiotics play a pivotal role in modulating the intestinal microbiota (Kaufmann et al., 2023). Preclinical studies have shown that supplementing probiotics has a significant improvement effect on ALD (Hong et al., 2015; Tian et al., 2015; Li et al., 2021a). Probiotic supplementation enhances populations of beneficial bacteria, such as *Bifidobacteria* and *Enterococci*, within the intestines while suppressing the growth of gram-negative bacteria, thereby reducing intestinal endotoxemia (Kim et al., 2018; Huang et al., 2019).

However, there is currently no consensus on the clinical utility of probiotics in patients with ALD. Therefore, the objective of this study is to systematically review published data regarding the clinical efficacy of probiotics in patients with ALD to provide valuable insights for evidence-based decision-making concerning probiotic intervention in ALD.

## 2 Materials and methods

### 2.1 Overview

This study is conducted in accordance with the PRISMA statement (Preferred Reporting Items for Systematic Reviews and Meta-analysis) (Page et al., 2021) and the Cochrane working manual, this study has been registered on PROSPERO (registration number: CRD42023472527).

### 2.2 Retrieval strategy

Relevant studies were identified through a comprehensive search of electronic databases, including PubMed, Embase, Web of Science, Cochrane Library, CNKI, VIP, Wanfang, and CBM. The investigation was limited from the inception of each database to October 15, 2023, and the literature search was not restricted to languages. From the perspective of evidence-based medicine, we need to collect all the research evidence of this topic as comprehensively as possible to minimize bias. Therefore, we conducted a comprehensive search on the above eight databases, and each electronic database has its unique advantages. For example, PubMed is one of the largest medical literature databases in the world, it covers many fields such as medicine, nursing and health care. Embase is a large biomedical database focused on drug research and pharmacology. Web of Science is a comprehensive academic literature database, which provides a global academic literature index and citation database in the fields of science, technology, medicine and social sciences. The Cochrane Library provides the latest clinical trial results and systematic reviews. In addition, within the limits of our resources, we also conducted searches of Chinese databases including CNKI, Wanfang, VIP and CBM. Subsequently, we conducted a meticulous review of the references cited in these articles to identify additional randomized controlled trials (RCTs). The search terms employed encompassed Medical Subject Headings (MeSH) terms and free words: “Alcoholic liver disease”, “Alcoholic fatty liver”, “Alcoholic hepatitis”, “Alcoholic liver fibrosis”, “Alcoholic cirrhosis”, “probiotics”, “probiotic”, “probiotic agent”, “ random* or placebo* or blind*”. Detailed search strategies are available in the Supplementary materials.

### 2.3 Inclusion and exclusion criteria

According to the PICOS principle, the inclusion criteria for articles consisted of the following: (1) inclusion of participants diagnosed with ALD, including alcoholic fatty liver disease, alcoholic hepatitis, alcoholic liver fibrosis, and alcoholic cirrhosis; (2) the intervention group was probiotic preparation. While the control group was treated with a placebo, other treatments, or no intervention; (3) including at least one of the following main indicators: alanine aminotransferase (ALT), aspartate aminotransferase (AST), γ-glutamyl transpeptidase (γ-GT), total bilirubin (TB), albumin (Alb). Additionally, tumor necrosis factor-α (TNF-α), interleukin-6 (IL-6), and intestinal flora are secondary outcome indicators; (4) utilization of an RCT study. Articles were excluded if they fulfilled the following conditions: ALD complicated with other chronic liver diseases; incomplete data or no clear outcome indicators; unable to obtain the full text; repeated publication.

### 2.4 Literature screening and data extraction

The literature was independently screened, and data were extracted by 2 researchers per the established protocol for preliminary duplicate inspection. Any discrepancies during this process were resolved through discussion or, if necessary, by a third-party arbitrator. The extracted data encompassed essential information from the included literature (such as first author, publication year, region, and title), sample size, age distribution, specific interventions employed in both experimental and control groups, treatment duration, and relevant outcome indicators.

### 2.5 Risk of bias evaluation

The quality of studies were assessed by Cochrane risk of bias tool Review Manager 5.3 (Nordic Cochran aa). The evaluations include the random sequence generation, allocation concealment, blinding of participants and personnel, blinding of outcome assessment, incomplete outcome data, selective reporting and other bias. The included studies were evaluated to three degrees including low, unclear, and high risk of bias.

### 2.6 Statistical analysis

The statistical analysis was conducted using RevMan 5.3 and Stata 14.2 software packages. Continuous variables were described using Weighted Mean Difference (WMD) or Standard Mean Deviation (SMD), along with their corresponding 95% confidence intervals (CI). A significance level of *α*=0.05 was employed, considering results statistically significant when *P*<0.05. The I² statistic was used to assess the heterogeneity within the model. If *P*>0.1 or I^2^<50%, it indicates an absence of heterogeneity, leading to a fixed effect model for analysis; otherwise, a random effects model was applied. The funnel plot served as a tool to evaluate publication bias, while Begg’s Test assessed the symmetry of said plot; *P* > 0.05 indicated a symmetrical funnel plot and no publication bias among included studies. Subgroup and sensitivity analyses were performed to assess the study’s robustness and explore potential sources of heterogeneity.

## 3 Results

### 3.1 Study selection

**Figure 1:**
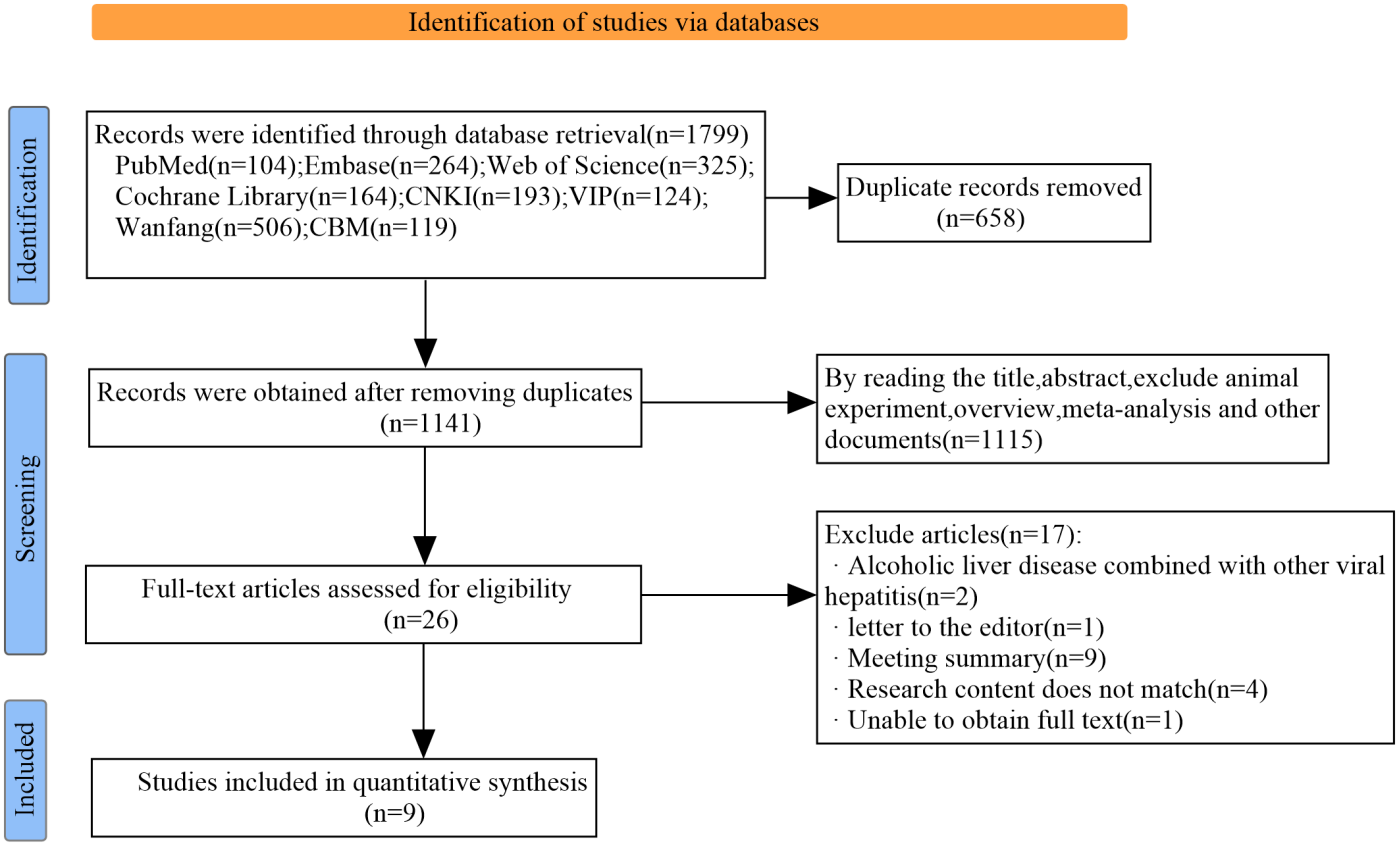
Flow chart of literature retrieval and screening.

A total of 1799 articles were retrieved through a computerized search, and 1141 articles were obtained after eliminating 658 duplicate reports. Following the assessment of titles, abstracts and full-text reading, animal experiments, systematic reviews, meta-analyses, and irrelevant studies were excluded. Ultimately, 9 original studies (Gupta et al., 2022); (Han et al., 2015); (Kirpich et al., 2008); (Koga et al., 2013); (Li et al., 2021b); (Stadlbauer et al., 2008); (Vatsalya et al., 2023); (Zhang et al., 2011); (Zhu and Wu, 2019)were included in the analysis. The specific screening process is illustrated in **Figure 1**.

### 3.2 Characteristics of included literature

**Table 1** presents the fundamental characteristics of the articles included, encompassing a total of 639 patients, with 355 in the experimental group and 284 in the control group, comprising 545 males and 94 females. Amongst the 9 original studies incorporated, 4 studies focused on alcoholic hepatitis and 2 studies on alcoholic cirrhosis; 1 study focused on alcoholic fatty liver disease, the other 2 studies did not clearly specify the stage of alcoholic liver disease in the participants.

**Table 1:** Basic characteristics of literature included in the literature.

First author(Year)	Place	Sample	Age	Sex(male:female)	Interventions		Duration(week)	Outcome
					Probiotics	control		
Gupta et al, 2022	Korea	44/45	50.8 ± 9.4	83:6	*Lacticaseibacillus rhamnosus* R0011and *Lactobacillus helveticus* R0052	Placebo	1	①②③④⑤⑥
Han et al, 2015	Korea	60/57	52.7 ± 11.3	75:42	*Lactobacillus subtilis*/*Streptococcus faecium*	Placebo	1	①②③④⑤⑥
Koga et al, 2013	Japan	18/19	53.3 ± 13.3	30:7	*Lactobacillus casei* Shirota YIT 9029	Placebo	4	①②③④⑤⑦
Li et al, 2021a	China	112/46	50.98 ± 5.1	158:0	*Lactobacillus casei*	Placebo	8	①②③④⑤⑦
Vatsalya et al, 2023	USA	24/22	44.6 ± 10.7	26:20	*Lactobacillus rhamnosus GG*	Placebo	4	①②④⑤
Zhu and Wu, 2019	China	40/40	47.05 ± 5.12	66:14	Polyene phosphatidylcholine capsules+ *Clostridium Butyricum* Tablets	Polyene phosphatidylcholine capsules	8	①②④⑥⑦
Kirpich et al, 2008	Russia	13/13	–	26:0	*Bifidobacterium bifidum* and *Lactobacillus plantarum 8PA3*	Vitamin	1	①②③④
Stadlbauer et al, 2008	U.K.	12/8	53.5 ± 18.3	15:5	*Lactobacillus casei* Shirota	Standard treatment	4	①④⑤
Zhang et al, 2011	China	32/34	45.75 ± 1.29	66:0	*Bifidobacterium,lactob-acillus* and *enterrococcus*	Standard treatment	1	①②③④

①ALT、②AST、③γ-GT、④TB、⑤ALB、⑥TNF-α、⑦IL-6.

### 3.3 Assessment of the quality of the included studies

**Figure 2:**
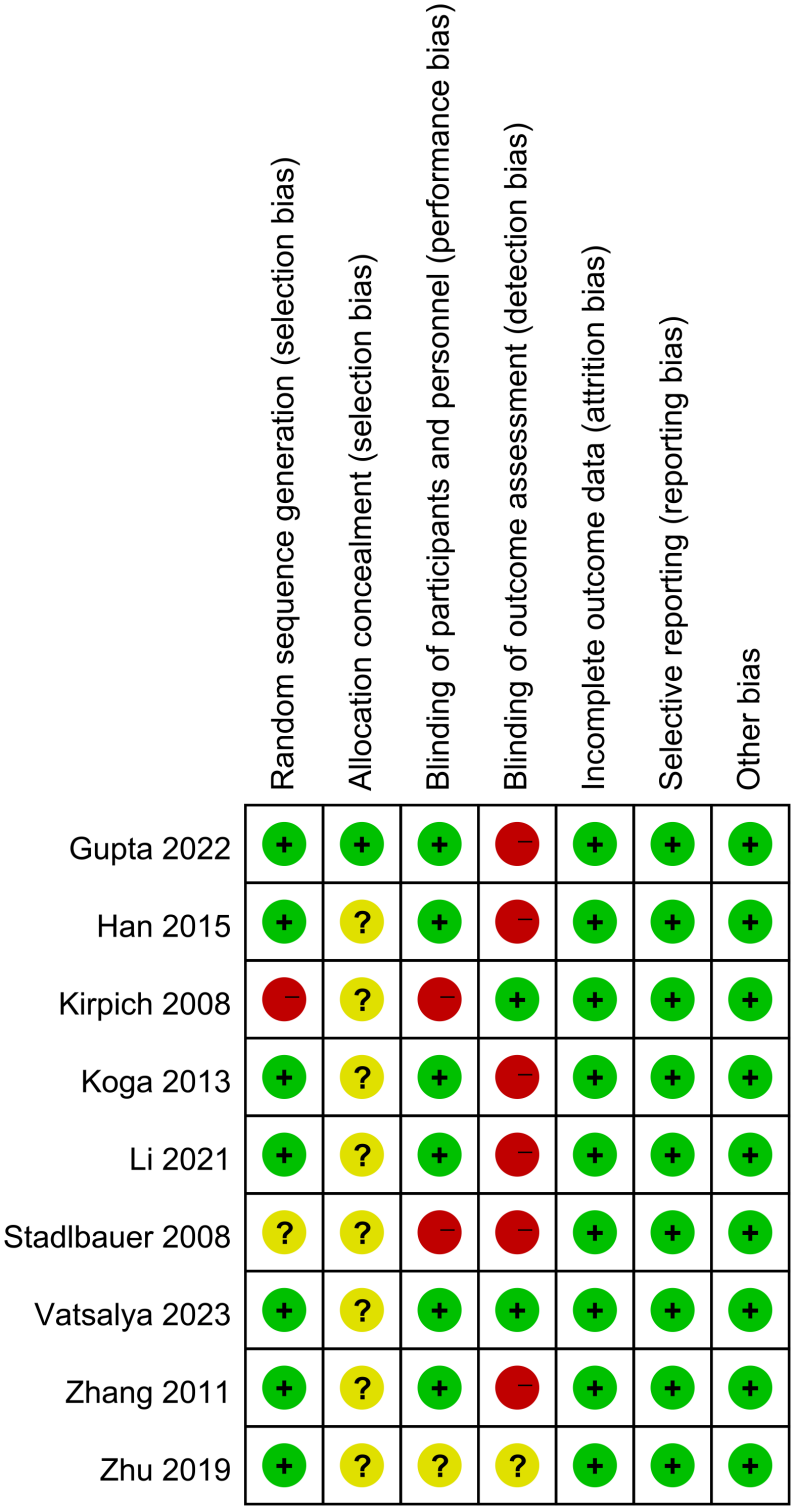
Risk of bias summary.

**Figure 3:**
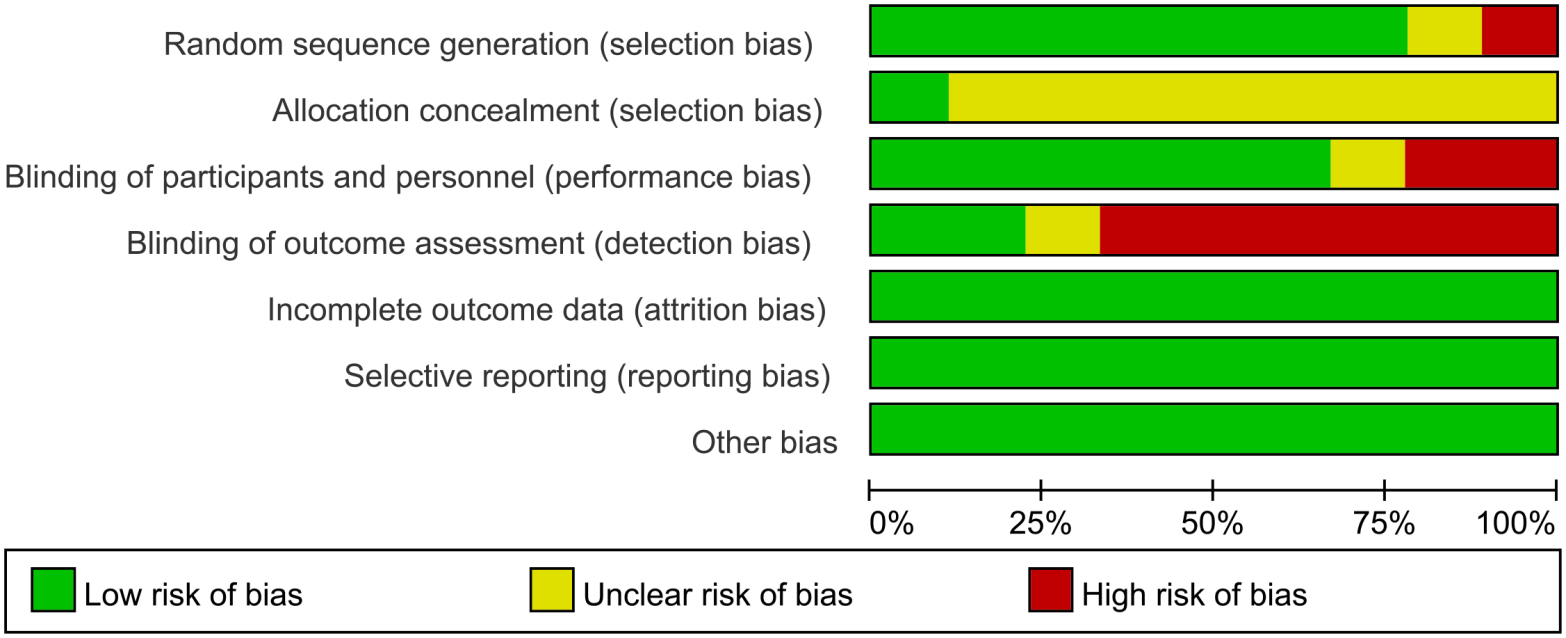
Risk of bias graph.

Among all included articles, 7 documents reported the generation of random sequences. Only 1 document mentioned allocation concealment, this indicated that there was selectivity bias in the included studies. 6 documents explained the blinding of researchers and subjects. Only 2 documents mentioned blinding evaluators, this suggests a greater risk of detection bias. All data were complete and selective report did not appear in all of the studies (**Figures 2**, **3**).

### 3.4 Meta-analysis

#### 3.4.1 Liver function

**Figure 4:**
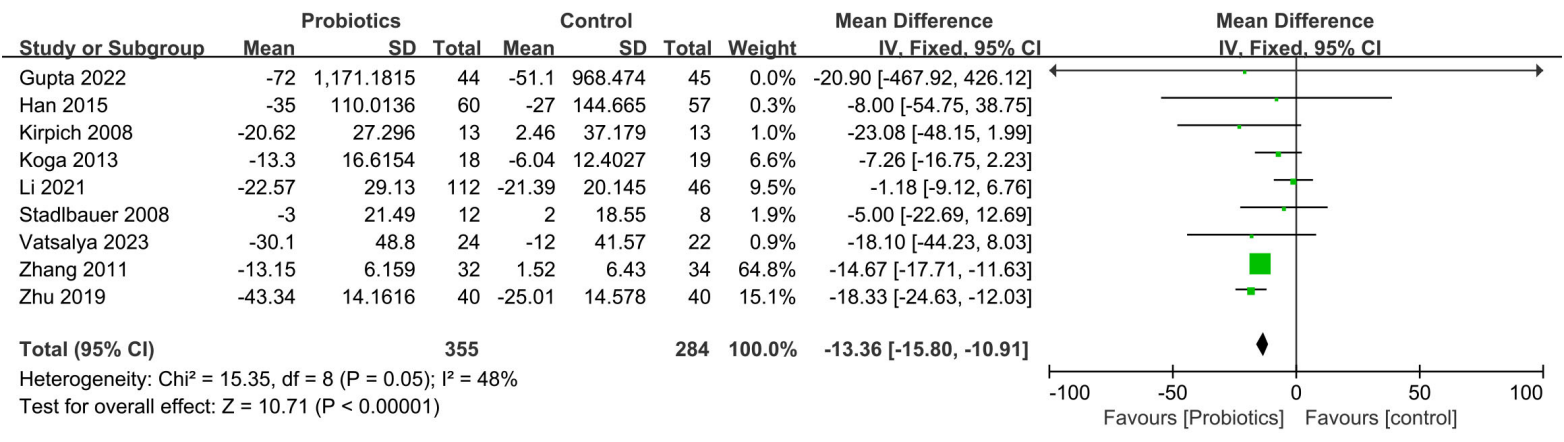
Forest plots of serum ALT level.

**Figure 5:**
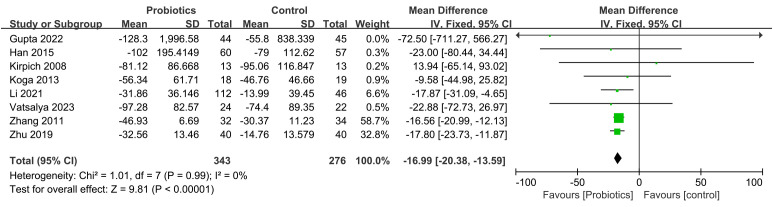
Forest plots of serum AST level.

**Figure 6:**
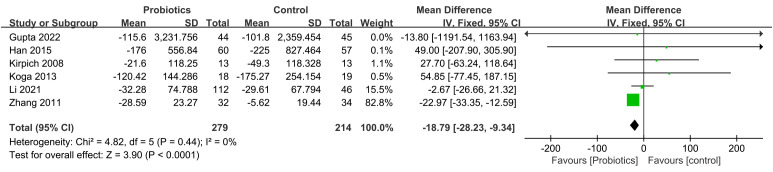
Forest plots of serum γ-GT level.

**Figure 7:**
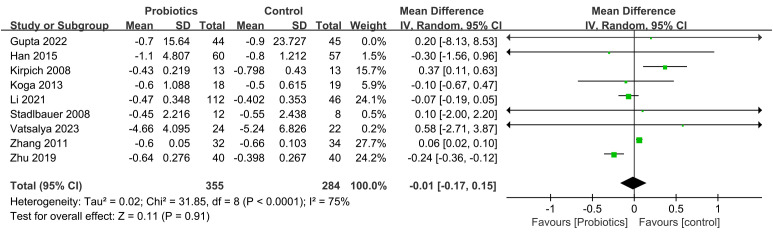
Forest plots of serum TB level.

**Figure 8:**
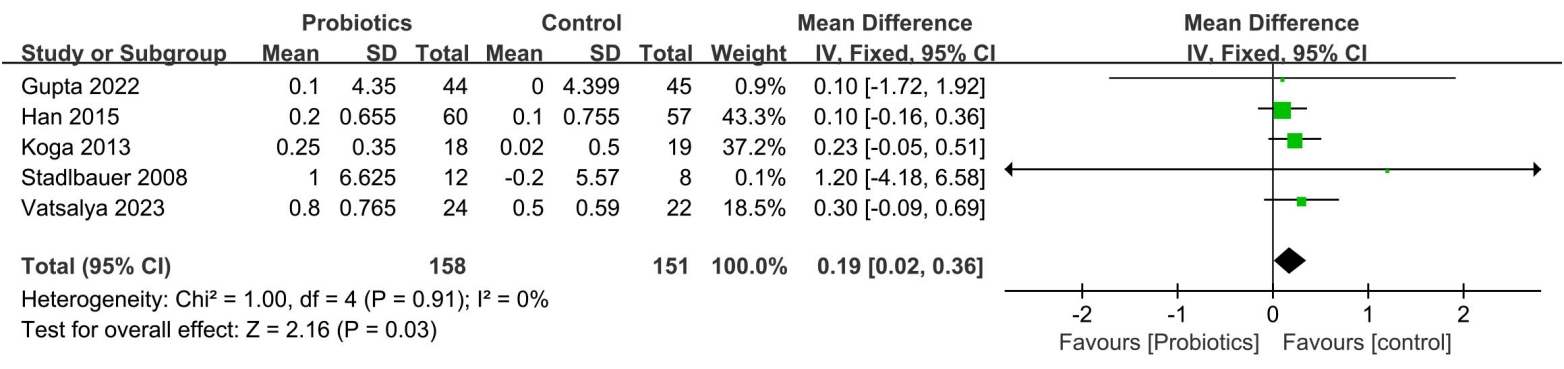
Forest plots of serum Alb level.

The impact of probiotic intervention on ALT was assessed in 9 studies. A fixed-effects model was used to combine the trials because there was statistical homogeneity for this outcome (I^2^= 48%). The combined effect size was found to be -13.36 (95% CI: -15.80, -10.91; *P*<0.00001), demonstrating that the probiotic intervention group exhibited a significant reduction in ALT levels compared to the control group among ALD patients (**Figure 4**). Additionally, 8 studies investigated the impact of the probiotic intervention on AST. The fixed effects model was employed for meta-analysis with I^2^ = 0%. The AST levels of patients with ALD were significantly decreased by treatment with probiotics (MD=-16.99, 95% CI: -20.38, -13.59; *P*<0.00001) (**Figure 5**). 6 studies reported on the impact of the probiotic intervention on γ-GT levels. Heterogeneity testing revealed an I^2^ = 0%, thus, a fixed-effects model was applied. The meta-analysis showed that compared to control group, the probiotics treatment significantly decreased the γ-GT levels (MD=-18.79, 95% CI: -28.23, -9.34; *P*<0.0001) (**Figure 6**). 9 studies examined the impact of the probiotic intervention on TB levels, however, substantial heterogeneity was observed with an I^2^ = 75% (**Figure 7**). We tried to conduct two subgroup analyzes based on differences in intervention time and stages of alcoholic liver disease, but neither could eliminate heterogeneity. 5 studies reported the effect of the probiotic intervention on Alb levels. Heterogeneity testing showed I^2^ = 0%; therefore, a fixed-effects model was used to pool data. The results indicated that compared to the control group, probiotic intervention significantly increased serum albumin levels in patients with ALD (MD=0.19, 95% CI:0.02,0.36; *P*=0.03) (**Figure 8**).

#### 3.4.2 Serum inflammatory factors

**Figure 9:**
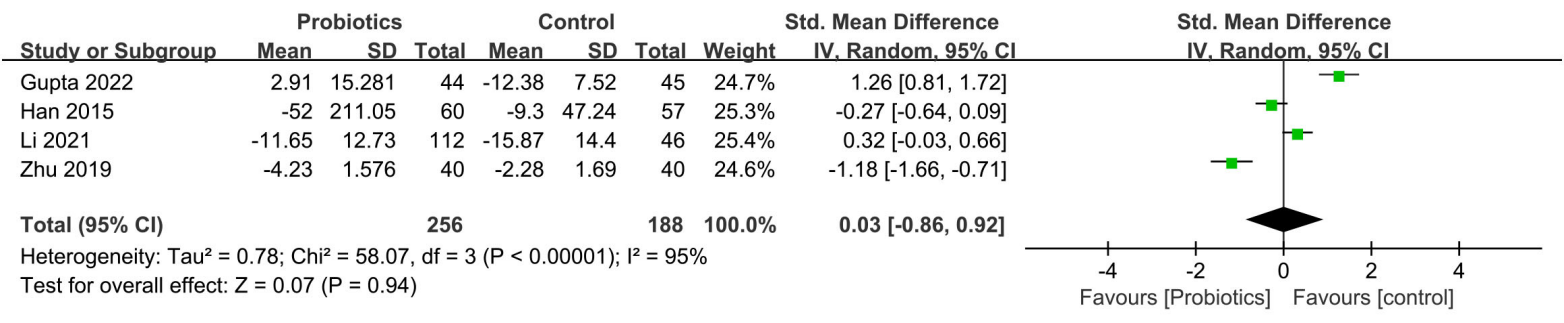
Forest plots of serum TNF- α level.

**Figure 10:**
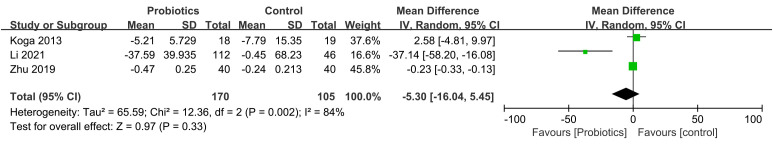
Forest plots of serum IL-6 level.

4 studies have reported on the impact of probiotic intervention on serum TNF-α levels in patients with ALD. The combined effect size was 0.03, (95% CI: -0.86, 0.92; *P*=0.94), the heterogeneity test results indicate a substantial heterogeneity (I^2^ = 94%). And we did not find significant changes in heterogeneity after a sensitivity analysis by sequentially excluding each study, which indicated the stability of the present random-effect model (**Figure 9**). 3 studies assessed the impact of probiotic intervention on serum IL-6 levels in ALD patients[MD=-5.30,(95% CI:-16.04,5.45), *P*=0.33], and a sensitivity analysis suggested that the Li’s study was the source of heterogeneity, which was reduced to 0% after excluding this article (**Figure 10**).

### 3.5 Intestinal microbiota

Considering the different assessment methods of intestinal flora among different studies, we did not combine this part of the data. As shown in **Table 2**, 7 studies reported the changes in intestinal flora after probiotic intervention. In general, after probiotic treatment, the proportion of Gram-negative bacilli such as *Escherichia coli* decreased, while the proportion of Gram-positive bacilli such as *Bifidobacterium* and *Lactobacillus* increased.

**Table 2:** Changes in intestinal flora.

	Disease type	Probiotic type	Changes in intestinal flora after probiotic supplementation
Gupta et al, 2022	Alcoholic hepatitis	*Lacticaseibacillus rhamnosus* R0011and *Lactobacillus helveticus* R0052	*Bacteroidetes*↑, *Proteobacteria* and *Fusobacteria*↓
Han et al, 2015	Alcoholic hepatitis	*Lactobacillus subtilis*/*Streptococcus faecium*	*Escherichia coli↓*
Kirpich et al, 2008	Alcoholic hepatitis	*Bifidobacterium bifidum* and *Lactobacillus plantarum 8PA3*	*Bifidobacteria* and *Lactobacilli*↑
Koga et al, 2013	Alcoholic cirrhosis	*Lactobacillus casei* Shirota YIT 9029	Obligate anerobic bacteria↑, *Enterobacteriaceae*↓
Li et al, 2021a	Alcoholic fatty liver	*Lactobacillus casei*	*Lactobacillus* and *Bifidobacterium*↑
Zhang et al, 2011	Alcoholic liver disease	*Bifidobacterium,lactob-acillus* and *enterrococcus*	*Bifidobacteria、Lactobacilli and enterococci*↑
Zhu and Wu, 2019	Alcoholic liver disease	*Clostridium Butyricum*	*Bifidobacteria、Lactobacilli*↑, *Escherichia coli*↓

"↑" indicates an increase; "↓" indicates a decrease.

### 3.6 Publication bias and sensitivity analysis

**Figure 11:**
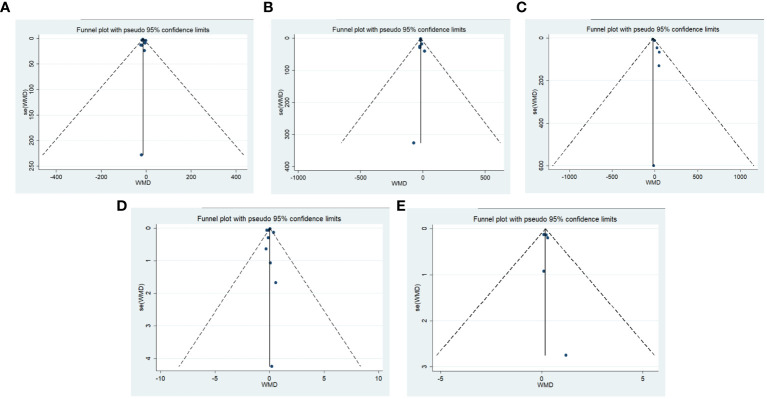
Publication bias funnel plot. **(A)** ALT, Egger’s Test result *P*=0.56; **(B)** AST, Egger’s Test result *P*=0.70; **(C)** γ-GT, Egger’s Test result *P*=0.09; **(D)** TB, Egger’s Test result *P*=0.66; **(E)** Alb, Egger’s Test result *P*=0.53.

Publication bias was tested using funnel plots and Egger’s regression test. The test results of ALT, AST, γ-GT, TB, Alb major outcome indicators showed that the *p* value of each major indicator was greater than 0.05, indicating that there was no significant publication bias (**Figure 11**). Sensitivity analysis was conducted on the major and demonstrated that even after systematically excluding individual studies for each indicator one by one, the integrated effect size remained within the 95% confidence interval of the original effect size. Consequently, these meta-analysis results can be considered relatively robust.

## 4 Discussion

This systematic review and meta-analysis aimed to investigate the efficacy of probiotic supplementation in individuals with ALD. Through analysis of 9 randomized controlled trials, we obtained valuable insights into the definitive effectiveness of probiotic preparations in patients with ALD. Specifically, meta-analysis results showed that supplementation with probiotic preparations significantly reduced serum ALT, AST, and γ-GT levels in ALD patients, and these findings suggest that probiotics are effective in alleviating liver inflammation in ALD patients. Furthermore, serum Alb levels increased following probiotic intervention, indicating a substantial improvement in liver synthesis function. However, no statistically significant difference was observed in bilirubin levels between the control group and the probiotic treatment group. In the clinical trials incorporated within this study, the probiotic intervention was administered for a duration ranging from 7 to 60 days. Given the short duration of the intervention included in the study, it is unlikely that bilirubin levels would have improved significantly in a short period of time. On the other hand, due to the different stages of alcoholic liver disease, different doses and types of probiotics taken, and a small number of included literature, there was significant heterogeneity in the results of TNF-a and IL-6. Therefore, in the future, we hope to have more studies to explore the impact of probiotics on the levels of inflammatory factors in ALD patients.

**Figure 12:**
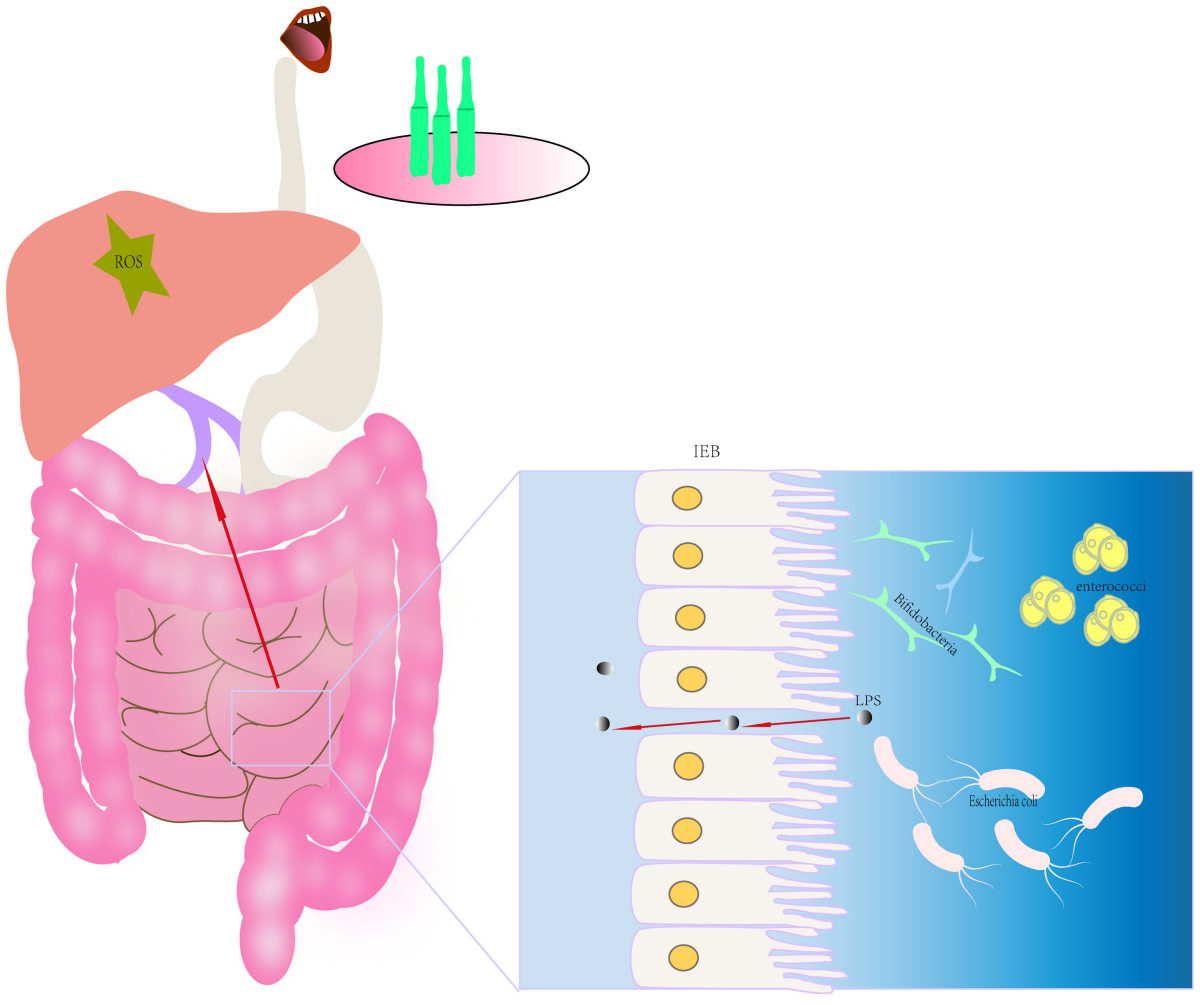
Schematic diagram of intestinal flora dysregulation.

Research has demonstrated that long-term ethanol exposure can lead to various liver diseases (Gu et al., 2019). When alcohol is ingested, 90% of it is absorbed by the small intestine and transported to the liver through the portal vein, and the remaining 10% is excreted through sweat, breath, and urine (Holford, 1987; Cederbaum, 2012). Both preclinical studies and clinical evidence suggest that alcohol consumption disrupts the composition and structure of the gut microbiome (Milosevic et al., 2019; Ranjbarian and Schnabl, 2023). Specifically, ethanol entering the intestine will cause an imbalance of the intestinal flora, increase the number of Gram-negative bacteria. In addition, alcohol will destroy the integrity of the intestinal mucosa and increase the intestinal permeability of macromolecules. Permeability, allowing endotoxins to pass from the intestine into the portal system (see **Figure 12**) (Chopyk and Grakoui, 2020). For instance, research conducted by Kirpich and his colleagues (Kirpich et al., 2008) demonstrated a significant reduction in *Bifidobacterium*, *Lactobacillus*, and *Enterococcus* in the feces of individuals with alcoholic patients, while the *Escherichia coli* exhibited an increasing trend. The study of Fang and his colleagues (Fang et al., 2023) found that ethanol consumption promotes the growth of Gram-negative bacteria such as Proteobacteria in the intestine and reduces the relative abundance of Firmicutes and Bacteroidetes. On the other hand, after ethanol enters the liver, it is oxidized to acetaldehyde in liver cells and then metabolized to acetic acid under the catalysis of acetaldehyde dehydrogenase (Cederbaum, 2012). Acetaldehyde induces liver damage by promoting glutathione consumption, ROS toxicity, and lipid peroxidation. Therefore, ethanol metabolism can induce direct biochemical reactions in liver cells, including the production of cytotoxic metabolites, reactive oxygen species (ROS) Accumulation and lipid peroxidation (Lamas-Paz et al., 2018).Gut-derived microbial lipopolysaccharide (LPS), a constituent of the outer membrane of Gram-negative bacteria, is known to play a pivotal role in the pathogenesis of AH (Szabo, 2015). Alcohol disrupts intestinal barrier function, thereby facilitating the translocation of LPS from the intestinal lumen into the portal vein and subsequently into the liver. Chronic alcohol exposure has been reported to sensitize Kupffer cells in the liver to LPS through the upregulation of microRNA-155 (Szabo et al., 2010), where LPS binds to Toll-like receptor 4 (TLR-4), ultimately activates various proinflammatory cytokines such as TNF-α (Tapper and Parikh, 2018). TNF-α, IL-1β, and IL-6 are important indicators reflecting the severity of inflammatory response and playing crucial roles in liver injury development. While TNF-α exerts direct cytotoxic effects, it can also cause microcirculatory disorders leading to hepatocyte necrosis, furthermore, it interacts with other inflammatory factors like IL-6 to further exacerbate liver damage (Kohn et al., 2002; Albano, 2008). It is worth noting that during liver injury, the levels of inflammatory cytokines increase and reach the intestine through circulation, disrupting the intestinal mucosal barrier. This phenomenon disrupts the balance of intestinal microbiota and forms a vicious cycle (Thurman et al., 1998).

The gut microbiome comprises a diverse array of bacterial strains and microorganisms that inhabit the stomach and intestinal tract (Jia et al., 2008), playing a crucial role in maintaining human homeostasis, metabolism, circulation, immunity, and hormone regulation (Qin et al., 2010; Schroeder and Bäckhed, 2016).The FAO (the United Nations Food and Agriculture Organization) and the WHO (the World Health Organization) defines probiotics as “live microorganisms that are beneficial to host health when ingested in moderate amounts” (Hill et al., 2014). Previous studies have demonstrated the potential role of probiotics in improving liver inflammation and regulating blood lipids in patients with non-alcoholic fatty liver disease (NAFLD) (Famouri et al., 2017; Ahn et al., 2019). Probiotics can effectively inhibit the occurrence of alcoholic liver disease, involving various potential mechanisms, including changes in gut microbiota, regulation of gut barrier function and immune response, reduction of endotoxin levels, and bacterial translocation (Chang et al., 2013; Vitetta et al., 2017; Mishra et al., 2023). It has been reported that supplementation of probiotics can inhibit the growth of pathogenic gram-negative bacteria, enhance phagocytosis activity, promote IgA secretion, and thus enhance cellular immune function (Zhang et al., 2010). Probiotics inhibit liver endotoxin levels by regulating gut flora, thereby reducing the production of pro-inflammatory markers (IL-6, TNF-α, IFN-γ, etc.) by down-regulating the expression of NF-kB (O’Sullivan, 2008). Recently, A meta-analysis based on animal studies has demonstrated that probiotic intervention can augment intestinal epithelial barrier (IEB) integrity, alleviate intestinal dysbiosis, and significantly ease liver function, blood lipids, inflammatory factors, and other indicators in ALD mice (Wang et al., 2022). Liu and his colleagues (Liu et al., 2023) have demonstrated that complex probiotics reduce LPS levels in the liver by upregulating mucus production and tight junction (TJ) protein expression, reversing acute alcohol-induced dysbiota and maintaining the integrity of the intestinal barrier.

Our analysis of the included original studies found that after probiotic intervention, the abundance of *Bifidobacteria* increased and Proteus decreased in the intestines of ALD patients, showing the potential role of probiotics treating ALD by regulating intestinal flora. However, due to the limited literature incorporating these indicators to date, we expect more high-quality research documents to support our findings in future investigations. Last but not least, all original studies emphasized strict abstinence from alcohol during the trial period. Even without probiotic treatment, liver function in the control group improved to varying degrees after alcohol abstinence, highlighting that alcohol abstinence is a basic requirement for ALD patients.

## 5 Strengths and limitations

To the best of our knowledge, this study represents the first meta-analysis investigating the clinical efficacy of probiotics in the treatment of ALD. Our findings demonstrate that probiotic intervention effectively improves liver function, reduces inflammatory factors, and regulates intestinal flora in patients with ALD. Moreover, no serious adverse events were reported in any of the included studies, indicating a higher level of safety associated with probiotic preparations. However, despite conducting systematic reviews and gaining valuable insights from this meta-analysis, it is essential to acknowledge certain limitations. Firstly, the number of included studies is relatively small, particularly concerning the composition of intestinal flora and relevant outcome indicators for inflammatory factors after probiotic intervention. This limitation restricts statistical power in these areas and underscores the need for further research. Secondly, heterogeneity exists among studies due to variations in intervention programs and durations. Therefore, future research should prioritize conducting high-quality RCTs with larger sample sizes to strengthen our conclusions.

## 6 Conclusion

In conclusion, probiotics have shown a good therapeutic effect in patients with ALD. It is speculated that the possible mechanism is to regulate intestinal flora and reduce the production of endotoxin, thereby improving liver function and reducing the body’s inflammatory response.

## Data availability statement

The original contributions presented in the study are included in the article/**Supplementary Material**. Further inquiries can be directed to the corresponding authors.

## Author contributions

S-YX: Data curation, Formal analysis, Investigation, Methodology, Writing – original draft, Writing – review & editing. G-SW: Formal analysis, Investigation, Writing – original draft. CL: Formal analysis, Investigation, Writing – original draft. WM: Validation, Visualization, Writing – review & editing. H-RL: Validation, Visualization, Writing – review & editing.

## References

[B1] Ahn S. B. Jun D. W. Kang B. K. Lim J. H. Lim S. Chung M. J. (2019). Randomized, double-blind, placebo-controlled study of a multispecies probiotic mixture in nonalcoholic fatty liver disease. Sci. Rep. 9, 5688. doi: 10.1038/s41598-019-42059-3 30952918 PMC6450966

[B2] Albano E. (2008). Oxidative mechanisms in the pathogenesis of alcoholic liver disease. Mol. Aspects Med. 29, 9–16. doi: 10.1016/j.mam.2007.09.004 18045675

[B3] Argemi J. Ventura-Cots M. Rachakonda V. Bataller R. (2020). Alcoholic-related liver disease: pathogenesis, management and future therapeutic developments. Rev. Esp Enferm Dig 112, 869–878. doi: 10.17235/reed.2020.7242/2020 33054302

[B4] Bajaj J. S. (2019). Alcohol, liver disease and the gut microbiota. Nat. Rev. Gastroenterol. Hepatol. 16, 235–246. doi: 10.1038/s41575-018-0099-1 30643227

[B5] Bull-Otterson L. Feng W. Kirpich I. Wang Y. Qin X. Liu Y. . (2013). Metagenomic analyses of alcohol induced pathogenic alterations in the intestinal microbiome and the effect of Lactobacillus rhamnosus GG treatment. PloS One 8, e53028. doi: 10.1371/journal.pone.0053028 23326376 PMC3541399

[B6] Cederbaum A. I. (2012). Alcohol metabolism. Clin. Liver Dis. 16, 667–685. doi: 10.1016/j.cld.2012.08.002 23101976 PMC3484320

[B7] Chang B. Sang L. Wang Y. Tong J. Zhang D. Wang B. (2013). The protective effect of VSL#3 on intestinal permeability in a rat model of alcoholic intestinal injury. BMC Gastroenterol. 13, 151. doi: 10.1186/1471-230X-13-151 24138544 PMC4016537

[B8] Chopyk D. M. Grakoui A. (2020). Contribution of the intestinal microbiome and gut barrier to hepatic disorders. Gastroenterology 159, 849–863. doi: 10.1053/j.gastro.2020.04.077 32569766 PMC7502510

[B9] Famouri F. Shariat Z. Hashemipour M. Keikha M. Kelishadi R. (2017). Effects of probiotics on nonalcoholic fatty liver disease in obese children and adolescents. J. Pediatr. Gastroenterol. Nutr. 64, 4133417. doi: 10.1097/MPG.0000000000001422 28230607

[B10] Fang C. Cheng J. Jia W. Xu Y. (2023). Akkermansia muciniphila ameliorates alcoholic liver disease in experimental mice by regulating serum metabolism and improving gut dysbiosis. Metabolites 13, 1057. doi: 10.3390/metabo13101057 37887381 PMC10608788

[B11] Galicia-Moreno M. Rosique-Oramas D. Medina-Avila Z. Álvarez-Torres T. Falcón D. Higuera-de la Tijera F. . (2016). Behavior of oxidative stress markers in alcoholic liver cirrhosis patients. Oxid. Med. Cell Longev 2016, 9370565. doi: 10.1155/2016/9370565 28074118 PMC5198187

[B12] Gu Z. Liu Y. Hu S. You Y. Wen J. Li W. . (2019). Probiotics for alleviating alcoholic liver injury. Gastroenterol. Res. Pract. 2019, 9097276. doi: 10.1155/2019/9097276 31263495 PMC6556793

[B13] Gupta H. Kim S. H. Kim S. K. Han S. H. Kwon H. C. Suk K. T. (2022). Beneficial Shifts in Gut Microbiota by Lacticaseibacillus rhamnosus R0011 and Lactobacillus helveticus R0052 in Alcoholic Hepatitis. Microorganisms 10, 1474. doi: 10.3390/microorganisms10071474 35889193 PMC9319967

[B14] Han S. H. Suk K. T. Kim D. J. Kim M. Y. Baik S. K. Kim Y. D. . (2015). Effects of probiotics (cultured Lactobacillus subtilis/Streptococcus faecium) in the treatment of alcoholic hepatitis: randomized-controlled multicenter study. Eur. J. Gastroenterol. Hepatol. 27, 1300–1306. doi: 10.1097/MEG.0000000000000458 26302024

[B15] Hill C. Guarner F. Reid G. Gibson G. R. Merenstein D. J. Pot B. . (2014). Expert consensus document. The International Scientific Association for Probiotics and Prebiotics consensus statement on the scope and appropriate use of the term probiotic. Nat. Rev. Gastroenterol. Hepatol. 11, 506–514. doi: 10.1038/nrgastro.2014.66 24912386

[B16] Holford N. H. (1987). Clinical pharmacokinetics of ethanol. Clin. Pharmacokinet. 13, 273–292. doi: 10.2165/00003088-198713050-00001 3319346

[B17] Hong M. Kim S. W. Han S. H. Kim D. J. Suk K. T. Kim Y. S. . (2015). Probiotics (Lactobacillus rhamnosus R0011 and acidophilus R0052) reduce the expression of toll-like receptor 4 in mice with alcoholic liver disease. PloS One 10, e0117451. doi: 10.1371/journal.pone.0117451 25692549 PMC4333821

[B18] Huang H. Lin Z. Zeng Y. Lin X. Zhang Y. (2019). Probiotic and glutamine treatments attenuate alcoholic liver disease in a rat model. Exp. Ther. Med. 18, 4733–4739. doi: 10.3892/etm.2019.8123 31777560 PMC6862500

[B19] Jia W. Li H. Zhao L. Nicholson J. K. (2008). Gut microbiota: a potential new territory for drug targeting. Nat. Rev. Drug Discovery 7, 123–129. doi: 10.1038/nrd2505 18239669

[B20] Kaufmann B. Seyfried N. Hartmann D. Hartmann P. (2023). Probiotics, prebiotics, and synbiotics in nonalcoholic fatty liver disease and alcohol-associated liver disease. Am. J. Physiol. Gastrointest Liver Physiol. 325, G42–g61. doi: 10.1152/ajpgi.00017.2023 37129252 PMC10312326

[B21] Kim W. G. Kim H. I. Kwon E. K. Han M. J. Kim D. H. (2018). Lactobacillus plantarum LC27 and Bifidobacterium longum LC67 mitigate alcoholic steatosis in mice by inhibiting LPS-mediated NF-κB activation through restoration of the disturbed gut microbiota. Food Funct. 9, 4255–4265. doi: 10.1039/C8FO00252E 30010169

[B22] Kirpich I. A. Solovieva N. V. Leikhter S. N. Shidakova N. A. Lebedeva O. V. Sidorov P. I. . (2008). Probiotics restore bowel flora and improve liver enzymes in human alcohol-induced liver injury: a pilot study. Alcohol 42, 675–682. doi: 10.1016/j.alcohol.2008.08.006 19038698 PMC2630703

[B23] Koga H. Tamiya Y. Mitsuyama K. Ishibashi M. Matsumoto S. Imaoka A. . (2013). Probiotics promote rapid-turnover protein production by restoring gut flora in patients with alcoholic liver cirrhosis. Hepatol. Int. 7, 767–774. doi: 10.1007/s12072-012-9408-x 26201812

[B24] Kohn G. Wong H. R. Bshesh K. Zhao B. Vasi N. Denenberg A. . (2002). Heat shock inhibits tnf-induced ICAM-1 expression in human endothelial cells via I kappa kinase inhibition. Shock 17, 91–97. doi: 10.1097/00024382-200202000-00002 11837795

[B25] Lamas-Paz A. Hao F. Nelson L. J. Vázquez M. T. Canals S. Gómez Del Moral M. . (2018). Alcoholic liver disease: Utility of animal models. World J. Gastroenterol. 24, 5063–5075. doi: 10.3748/wjg.v24.i45.5063 30568384 PMC6288648

[B26] Li X. Liu Y. Guo X. Ma Y. Zhang H. Liang H. (2021b). Effect of Lactobacillus casei on lipid metabolism and intestinal microflora in patients with alcoholic liver injury. Eur. J. Clin. Nutr. 75, 1227–1236. doi: 10.1038/s41430-020-00852-8 33514869 PMC8352779

[B27] Li H. Shi J. Zhao L. Guan J. Liu F. Huo G. . (2021a). Lactobacillus plantarum KLDS1.0344 and Lactobacillus acidophilus KLDS1.0901 Mixture Prevents Chronic Alcoholic Liver Injury in Mice by Protecting the Intestinal Barrier and Regulating Gut Microbiota and Liver-Related Pathways. J. Agric. Food Chem. 69, 183–197. doi: 10.1021/acs.jafc.0c06346 33353302

[B28] Liu H. Kang X. Yang X. Yang H. Kuang X. Ren P. . (2023). Compound probiotic ameliorates acute alcoholic liver disease in mice by modulating gut microbiota and maintaining intestinal barrier. Probiotics Antimicrob. Proteins 15, 185–201. doi: 10.1007/s12602-022-10005-x 36456838

[B29] Llopis M. Cassard A. M. Wrzosek L. Boschat L. Bruneau A. Ferrere G. . (2016). Intestinal microbiota contributes to individual susceptibility to alcoholic liver disease. Gut 65, 830–839. doi: 10.1136/gutjnl-2015-310585 26642859

[B30] Mellinger J. L. Shedden K. Winder G. S. Tapper E. Adams M. Fontana R. J. . (2018). The high burden of alcoholic cirrhosis in privately insured persons in the United States. Hepatology 68, 872–882. doi: 10.1002/hep.29887 29579356

[B31] Milosevic I. Vujovic A. Barac A. Djelic M. Korac M. Radovanovic Spurnic A. . (2019). Gut-liver axis, gut microbiota, and its modulation in the management of liver diseases: A review of the literature. Int. J. Mol. Sci. 20, 395. doi: 10.3390/ijms20020395 30658519 PMC6358912

[B32] Mishra G. Singh P. Molla M. Yimer Y. S. Dinda S. C. Chandra P. . (2023). Harnessing the potential of probiotics in the treatment of alcoholic liver disorders. Front. Pharmacol. 14. doi: 10.3389/fphar.2023.1212742 PMC10287977 37361234

[B33] O’Sullivan D. J. (2008). Genomics can advance the potential for probiotic cultures to improve liver and overall health. Curr. Pharm. Des. 14, 1376–1381. doi: 10.2174/138161208784480234 18537660

[B34] Page M. J. McKenzie J. E. Bossuyt P. M. Boutron I. Hoffmann T. C. Mulrow C. D. . (2021). The PRISMA 2020 statement: an updated guideline for reporting systematic reviews. Bmj 372, n71. doi: 10.1136/bmj.n71 33782057 PMC8005924

[B35] Qin J. Li R. Raes J. Arumugam M. Burgdorf K. S. Manichanh C. . (2010). A human gut microbial gene catalogue established by metagenomic sequencing. Nature 464, 59–65. doi: 10.1038/nature08821 20203603 PMC3779803

[B36] Ranjbarian T. Schnabl B. (2023) Gut microbiome-centered therapies for alcohol-associated liver disease. Semin. Liver. Dis. 43, 311–322. doi: 10.1055/a-2145-7331 37527781

[B37] Schroeder B. O. Bäckhed F. (2016). Signals from the gut microbiota to distant organs in physiology and disease. Nat. Med. 22, 1079–1089. doi: 10.1038/nm.4185 27711063

[B38] Siddiqi F. A. Sajja K. C. Latt N. L. (2020). Current management of alcohol-associated liver disease. Gastroenterol. Hepatol. (N Y) 16, 561–570. 34035691 PMC8132623

[B39] Singal A. K. Bataller R. Ahn J. Kamath P. S. Shah V. H. (2018). ACG clinical guideline: alcoholic liver disease. Am. J. Gastroenterol. 113, 175–194. doi: 10.1038/ajg.2017.469 29336434 PMC6524956

[B40] Stadlbauer V. Mookerjee R. P. Hodges S. Wright G. A. Davies N. A. Jalan R. (2008). Effect of probiotic treatment on deranged neutrophil function and cytokine responses in patients with compensated alcoholic cirrhosis. J. Hepatol. 48, 945–951. doi: 10.1016/j.jhep.2008.02.015 18433921

[B41] Szabo G. (2015). Gut-liver axis in alcoholic liver disease. Gastroenterology 148, 30–36. doi: 10.1053/j.gastro.2014.10.042 25447847 PMC4274189

[B42] Szabo G. Bala S. Petrasek J. Gattu A. (2010). Gut-liver axis and sensing microbes. Dig Dis. 28, 737–744. doi: 10.1159/000324281 21525758 PMC3211517

[B43] Tapper E. B. Parikh N. D. (2018). Mortality due to cirrhosis and liver cancer in the United State-2016: observational study. Bmj 362, k2817. doi: 10.1136/bmj.k2817 30021785 PMC6050518

[B44] Thurman R. G. Bradford B. U. Iimuro Y. Knecht K. T. Arteel G. E. Yin M. . (1998). The role of gut-derived bacterial toxins and free radicals in alcohol-induced liver injury. J. Gastroenterol. Hepatol. 13, S39–s50. doi: 10.1111/jgh.1998.13.s1.39 28976690

[B45] Tian F. Chi F. Wang G. Liu X. Zhang Q. Chen Y. . (2015). Lactobacillus rhamnosus CCFM1107 treatment ameliorates alcohol-induced liver injury in a mouse model of chronic alcohol feeding. J. Microbiol. 53, 856–863. doi: 10.1007/s12275-015-5239-5 26626356

[B46] Vatsalya V. Feng W. Kong M. Hu H. Szabo G. McCullough A. . (2023). The beneficial effects of lactobacillus GG therapy on liver and drinking assessments in patients with moderate alcohol-associated hepatitis. Am. J. Gastroenterol. 118, 1457–1460. doi: 10.14309/ajg.0000000000002283 37040544 PMC10524173

[B47] Vitetta L. Saltzman E. T. Thomsen M. Nikov T. Hall S. (2017). Adjuvant probiotics and the intestinal microbiome: enhancing vaccines and immunotherapy outcomes. Vaccines (Basel) 5, 50. doi: 10.3390/vaccines5040050 29232932 PMC5748616

[B48] Wang Q. Shi J. Zhao M. Ruan G. Dai Z. Xue Y. . (2022). Microbial treatment of alcoholic liver disease: A systematic review and meta-analysis. Front. Nutr. 9. doi: 10.3389/fnut.2022.1054265 PMC9719948 36479298

[B49] World Health Organization (2018). Global status report on alcohol and health 2018 (Geneva: World Health Organization).

[B50] Zhang W. Gu Y. Chen Y. Deng H. Chen L. Chen S. . (2010). Intestinal flora imbalance results in altered bacterial translocation and liver function in rats with experimental cirrhosis. Eur. J. Gastroenterol. Hepatol. 22, 1481–1486. doi: 10.1097/MEG.0b013e32833eb8b0 20739895

[B51] Zhang D. Hao X. Yang T. Lou Y. (2011). The therapeutic role of liver combined Bifidobacterium,lactobacillus and enterococcus in alcoholic liver disease. China Med. 06, 555–558. doi: 10.3760/cma.j.issn.1673-4777.2011.05.020

[B52] Zhu H. Wu J. S. (2019). Influence of Clostridium Butyricum Tablets Combined with Polyene Phosphatidyl Choline on intestinal flora and inflammatory factors of patients with alcoholic liver disease. Chin. J. Microecology 31, 82–85. doi: 10.13381/j.cnki.cjm.201901018

