# Supplementary material for: Clinical efficacy of probiotics in the treatment of alcoholic liver disease: a systematic review and meta-analysis
Source: Front Cell Infect Microbiol. 2024 Mar 12;14:1358063. doi: 10.3389/fcimb.2024.1358063 (PMC10964906; doi:10.3389/fcimb.2024.1358063)
Supplement: Supplementary file 1 [file DataSheet_1.docx]

Supplementary Material

# Supplementary Tables

## PubMed database search query

| Search number | Query | Results |
| --- | --- | --- |
| 9 | ((("Liver Diseases, Alcoholic"[Mesh]) OR ((alcoholic and (liver disease* or steatosis or fibrosis or hepatitis or cirrhosis)))) AND (("Probiotics"[Mesh]) OR ((Probiotics[Title/Abstract]) OR (Probiotic[Title/Abstract])))) AND ((random* or placebo* or blind*)) | 104 |
| 8 | (random* or placebo* or blind*) | 1,919,314 |
| 7 | (("Liver Diseases, Alcoholic"[Mesh]) OR ((alcoholic and (liver disease* or steatosis or fibrosis or hepatitis or cirrhosis)))) AND (("Probiotics"[Mesh]) OR ((Probiotics[Title/Abstract]) OR (Probiotic[Title/Abstract]))) | 472 |
| 6 | ("Probiotics"[Mesh]) OR ((Probiotics[Title/Abstract]) OR (Probiotic[Title/Abstract])) | 44,012 |
| 5 | (Probiotics[Title/Abstract]) OR (Probiotic[Title/Abstract]) | 39,963 |
| 4 | "Probiotics"[Mesh] | 24,816 |
| 3 | ("Liver Diseases, Alcoholic"[Mesh]) OR ((alcoholic and (liver disease* or steatosis or fibrosis or hepatitis or cirrhosis))) | 46,958 |
| 2 | (alcoholic and (liver disease* or steatosis or fibrosis or hepatitis or cirrhosis)) | 44,029 |
| 1 | "Liver Diseases, Alcoholic"[Mesh] | 16,123 |

## Embase database search query

| No. | Query | Results |
| --- | --- | --- |
| #9 | #7 AND #8 | 264 |
| #8 | random* OR blind* OR placebo* | 2719607 |
| #7 | #3 AND #6 | 950 |
| #6 | #4 OR #5 | 62663 |
| #5 | 'probiotic agent':ab,ti OR probiotic:ab,ti OR probiotics:ab,ti | 46538 |
| #4 | 'probiotic agent'/exp | 53701 |
| #3 | #1 OR #2 | 86882 |
| #2 | ('alcoholic'/exp OR alcoholic) AND (('liver'/exp OR liver) AND disease* OR 'steatosis'/exp OR steatosis OR 'fibrosis'/exp OR fibrosis OR 'hepatitis'/exp OR hepatitis OR 'cirrhosis'/exp OR cirrhosis) | 77848 |
| #1 | 'alcohol liver disease'/exp | 32208 |

## Web of Science database search query

| No. | Query | Results |
| --- | --- | --- |
| 74 | TS=(alcoholic and (liver disease* or steatosis or fibrosis or hepatitis or cirrhosis)) and Preprint Citation Index | 99193 |
| 83 | ((TS=("probiotic agent")) OR TS=(probiotic)) OR TS=(probiotics) and Preprint Citation Index | 101212 |
| 85 | #83 AND #74 and Preprint Citation Index | 1072 |
| 88 | TS=(random* or placebo* or blind*) and Preprint Citation Index | 4339264 |
| 92 | #88 AND #85 and Preprint Citation Index | 325 |

## Cochrane Library database search query

| #1 | MeSH descriptor: [Liver Diseases, Alcoholic] explode all trees | MeSH | 535 |
| --- | --- | --- | --- |
| #2 | alcoholic and (liver disease* or steatosis or fibrosis or hepatitis or cirrhosis) | Limits | 4931 |
| #3 | #1 OR #2 | Limits | 4937 |
| #4 | MeSH descriptor: [Probiotics] explode all trees | MeSH | 3092 |
| #5 | (Probiotic):ti,ab,kw OR (probiotic agent):ti,ab,kw OR (probiotics):ti,ab,kw | Limits | 9694 |
| #6 | #4 OR #5 | Limits | 9811 |
| #7 | #3 AND #6 | Limits | 164 |

## CNKI, VIP, Wanfang, and CBM database search query

| Database | Query | Results |
| --- | --- | --- |
| CNKI | ((((((subject%='alcoholic liver disease' or title%='alcoholic liver disease')= OR (subject%='alcoholic fatty liver disease' or title%='alcoholic fatty liver disease'))= OR (subject%='alcoholic hepatitis' or title%='alcoholic hepatitis ')) OR (subject%='alcoholic liver fibrosis' or subject %='alcoholic liver fibrosis' or title%='alcoholic liver fibrosis' or title%='alcoholic liver fibrosis ')) OR (subject%='alcoholic cirrhosis' or title%='alcoholic cirrhosis')) AND (subject%='probiotics' or title%='probiotics')) | 193 |
| VIP | (M=(alcoholic liver disease or alcoholic fatty liver disease or alcoholic hepatitis or alcoholic liver fibrosis or alcoholic cirrhosis) or R=(alcoholic liver disease or alcoholic fatty liver disease or alcoholic hepatitis or alcoholic liver fibrosis or alcoholic cirrhosis) ) AND (M=(probiotics) or R=(probiotics)) | 124 |
| Wanfang | subject: (alcoholic liver disease or alcoholic fatty liver disease or alcoholic hepatitis or alcoholic liver fibrosis or alcoholic cirrhosis) and subject: (probiotics) | 506 |
| CBM | ('Alcoholic liver disease' OR 'Alcoholic fatty liver disease' OR 'Alcoholic hepatitis' OR 'Alcoholic liver fibrosis' OR 'Alcoholic cirrhosis') AND (' Probiotics') | 119 |
